# Identification of bacterial pathogens in cultured fish with a custom peptide database constructed by matrix-assisted laser desorption/ionization time-of-flight mass spectrometry (MALDI-TOF MS)

**DOI:** 10.1186/s12917-020-2274-1

**Published:** 2020-02-11

**Authors:** Patharapol Piamsomboon, Janthima Jaresitthikunchai, Tran Quang Hung, Sittiruk Roytrakul, Janenuj Wongtavatchai

**Affiliations:** 1 Department of Veterinary Medicine, Faculty of Veterinary Sciences, Chulalongkorn University, Bangkok, Thailand; 2 Proteomics Research Laboratory, National Center for Genetic Engineering and Biotechnology, Pathum Thani, Thailand; 3 University of South Bohemia in Ceske Budejovice, Faculty of Fisheries and Protection of Waters, South Bohemian Research Center of Aquaculture and Biodiversity of Hydrocenoses, Zátiší 728/II, 389 25, Vodňany, Czech Republic

**Keywords:** Mass spectrometry, Proteomic, Fish disease, Biotyper

## Abstract

**Background:**

The majority of infectious diseases of cultured fish is caused by bacteria. Rapid identification of bacterial pathogens is necessary for immediate management. The present study developed a custom Main Spectra Profile (MSP) database and validate the method using matrix-assisted laser desorption/ionization time-of-flight mass spectrometry (MALDI-TOF MS) for rapid identification of fish bacterial pathogens. *Streptococcus agalactiae, Streptococcus iniae, Aeromonas hydrophila*, *Aeromonas veronii,* and *Edwardsiella tarda* obtained from diseased fish were used as representative bacterial pathogens in this study. Bacterial peptides were extracted to create a Main Spectra Profile (MSP), and the MSPs of each bacterial species was added into the MALDI Biotyper database. Fifteen additional isolates of each bacterial species were tested to validate the utilized technique.

**Results:**

The MSPs of all field isolates were clearly distinguishable, and the MSPs of the same species were clustered together. The identification methodology was validated with 75 bacterial isolates. The reliability and specificity of the method were determined with MALDI Biotyper log score values and matching results with 16 s rDNA sequencing. The species identification using the public MALDI Biotyper library (Bruker MALDI Biotyper) showed unreliable results (log score < 2.000) with 42.67% matching result with the reference method. In contrast, accurate identification was obtained when using the custom-made database, giving log score > 2.115, and a 100% matching result.

**Conclusion:**

This study demonstrates an effective identification of fish bacterial pathogens when a complete custom-made MSP database is applied. Further applications require a broad, well-established database to accommodate prudent identification of many fish bacterial pathogens by MALDI-TOF MS.

## Background

Bacterial pathogens are a major etiology of infectious diseases of cultured fish [1]. Among those bacteria, *Streptococcus* spp*., Aeromonas* spp*.*, and *Edwardsiella* spp., are commonly found in several important aquaculture species, such as the Asian catfish *Clarias batrachus* [2], barramundi *Lates calcarifer* [3], and Nile tilapia *Oreochromis niloticus* [4]. In many cases of bacterial infection, clinical signs and lesions are not obviously apparent and may mislead the diagnosis. Therefore, identification of disease-causing bacterial species is necessary in order to carry out proper disease management.

Conventional microbiology techniques, including morphological, physiological and biochemical tests, and molecular techniques based on 16S rDNA sequencing, are the gold standard for bacterial species identification [5]. However, these techniques require a substantial amount of time and expensive reagents [6]. In recent developments of mass spectrometry (MS), matrix-assisted laser desorption/ionization time-of-flight mass spectrometry (MALDI-TOF MS) has been implemented in human and veterinary medicine as an alternative diagnostic tool with increasing popularity due to its quickness, simplicity, cost-effectiveness, and strong discriminating power [7, 8]. The MALDI-TOF MS detects mass signals from bacterial proteins or peptides and determines their unique mass spectra or peptide mass fingerprints (PMFs). The obtained PMFs are then compared with reference bacterial strains in the public proteomics/genomics databases, or in a dedicated mass spectra library (library based approach) [9]. These mass spectra libraries are able to differentiate the bacteria to their genus, species or sub-group levels subject to sufficient pre-existing reference strains in the database [10].

The MALDI-TOF MS approach has been adopted as a routine diagnostic tool for human medicine [11] and has also been widely evaluated for its ability to differentiate bacterial species of veterinary and public health importance. For example, *S. equi* at the subspecies level [8], *Streptococcus* species isolated from diseased pigs [12], pathogenic Gram-negative bacteria in seafood [13] and Aeromonas species found in a drinking water system [14] have been assessed. In fish, MALDI-TOF was evaluated for the rapid identification of Gram-positive bacterial pathogens, including *S. agalactiae*, *Lactococcus garvieae*, *S. iniae*, and *S. dysgalactiae* isolated from Nile tilapia [15] and *S. iniae* isolated from the olive flounder *Paralichthys olivaceus* [16]. These studies found that the public database, the Bruker MALDI Biotyper library, was insufficient for identifying bacterial species isolated from fish with MALDI-TOF MS.

Therefore, the present study aims to develop a custom Main Spectra Profile (MSP) database and validate the method using MALDI-TOF MS for a rapid and accurate identification of *S. agalactiae, S. iniae, A. hydrophila, A. veronii* and *E. tarda* isolated from economically important fish species.

## Results

### Maldi-Tof Ms. for bacterial species differentiation

**Fig. 1:**
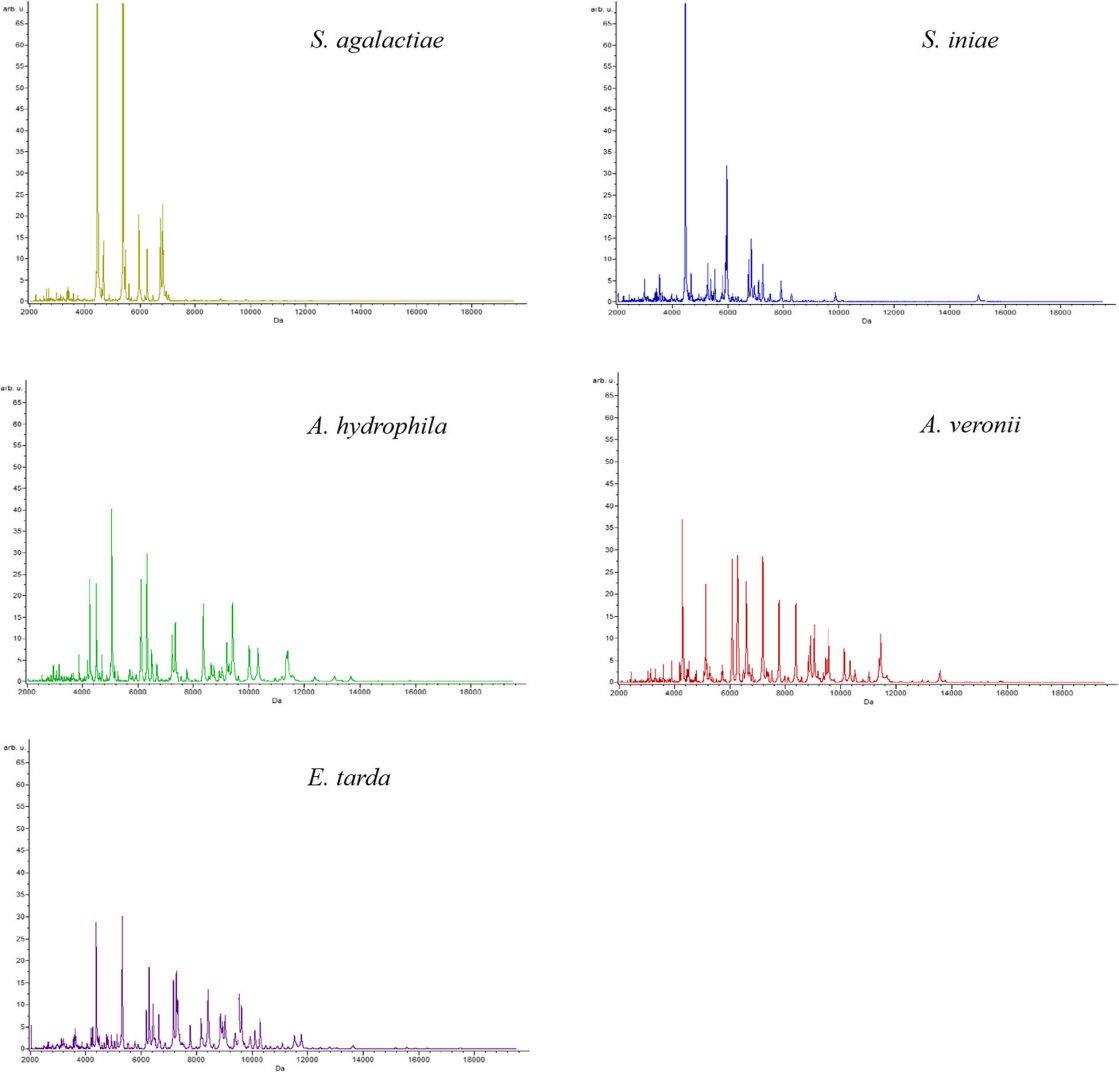
Mass peptide fingerprints of five bacterial pathogens (*S. agalactiae, S. iniae, A. hydrophila*, *A. veronii* and *E. tarda*) isolated from cultured fish

**Fig. 2:**
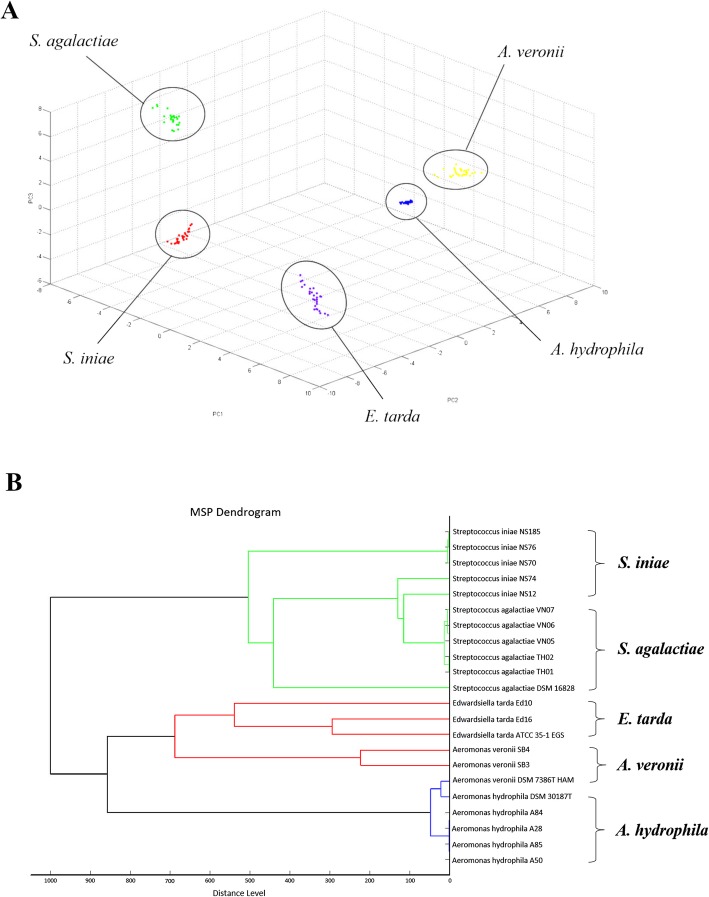
Cluster analysis of the five bacterial pathogens (*S. agalactiae, S. iniae, A. hydrophila*, *A. veronii* and *E. tarda*) isolated from cultured fish. **a** The 3D-PCA scatterplot representing clusters of each species (dashed circles) and the (**b**) MSP dendrogram of the representative bacterial isolates analyzed in the present study with the reference ATCC strains

The high reliability of the MALDI-TOF MS was indicated by the obtained 100% recognition capabilities and by the cross-validation values of 87.8, 97.1, 100, 100, and 100% for *S. agalactiae, S. iniae. A. hydrophila, A. veronii*, and *E. tarda,* respectively. The five bacterial species showed distinguishing spectral peaks ranging between 2000 and 15,000 Da (Fig. 1). The three-dimensional principal component analysis (3D-PCA) scatterplot presented clearly distinguishable clusters, each cluster presented in the 3D-PCA scatterplot (Fig. 2a) indicates MSPs or distinctive peptide fingerprint of the bacterial species. Bacterial isolates of the same species were grouped within the same clade of MSP dendrogram (Fig. 2b).

### Bacterial identification with MALDI Biotyper

The 75 tested isolates, when blasted with the reference strains available in the Bruker database, gave no-reliable identification for 23 isolates (log score 1.432–1.669), probable genus-level identification for 42 isolates (log score 1.707–1.998), and secure genus-level identification for 10 isolates (log score 2.018–2.254). No species-level identification was obtained, particularly for *S. iniae* since it is not available in the Bruker database (Table 1). Differently, all 75 tested bacterial isolates yielded accurate genus-level identification with a custom MSP database, 66 isolates were identified highly probable species-level identification (log score 2.301–3.001), and 9 isolates were identified a probable species-level identification (log score 2.115–2.264). Repeatability of the method was considered with ≤10% deviations of log scores (Table 2). For specificity, a custom MSP database provided 100% match with 16 s rDNA sequencing, while the Bruker database yielded matching result of 42.67% (32 out of 75 isolates).

**Table 1:** Method validation of the MALDI Biotyper of five bacterial pathogens showing the matching results with the 16 s rDNA sequencing, custom MSP and Bruker MSP database. The identity was calculated against NCBI database

Isolate number	Source	16S rDNA sequencing		MALDI-TOF			
				Custom MSP database		Bruker MSP database	
		Organism best match	% identity	Organism best match	Log score	Organism best match	Log score
*Streptococcus spp*							
S147-J^a^	Nile tilapia	*S. agalactiae*	100	*S. agalactiae*	2.999	*S. parauberis*	1.877
S183-J^a^	Red tilapia	*S. agalactiae*	100	*S. agalactiae*	2.897	*S. parauberis*	1.665
S187-J^a^	Nile tilapia	*S. agalactiae*	99	*S. agalactiae*	2.988	*S. agalactiae*	1.545
S190-J^a^	Red tilapia	*S. agalactiae*	99	*S. agalactiae*	2.856	*S. agalactiae*	1.998
SV1/1-J^a^	Nile tilapia	*S. agalactiae*	100	*S. agalactiae*	2.945	*S. agalactiae*	2.019
S71	Red tilapia	*S. agalactiae*	99	*S. agalactiae*	2.244	*S. urinalis*	1.666
S96	Red tilapia	*S. agalactiae*	100	*S. agalactiae*	2.377	*S. agalactiae*	1.756
S100	Nile tilapia	*S. agalactiae*	99	*S. agalactiae*	2.442	*S. agalactiae*	1.582
S101	Nile tilapia	*S. agalactiae*	99	*S. agalactiae*	2.375	*S. parauberis*	1.829
S102	Nile tilapia	*S. agalactiae*	98	*S. agalactiae*	2.544	*S. parauberis*	1.876
S183	Red tilapia	*S. agalactiae*	100	*S. agalactiae*	2.388	*S. agalactiae*	1.716
S184	Red tilapia	*S. agalactiae*	100	*S. agalactiae*	2.591	*S. agalactiae*	1.749
S191	Red tilapia	*S. agalactiae*	99	*S. agalactiae*	2.347	*S. urinalis*	1.564
S195	Nile tilapia	*S. agalactiae*	97	*S. agalactiae*	2.455	*S. agalactiae*	1.632
S198	Nile tilapia	*S. agalactiae*	99	*S. agalactiae*	2.544	*S. agalactiae*	1.908
NS12-J^a^	Red tilapia	*S. iniae*	100	*S. iniae*	2.898	*S. agalactiae*	1.432
NS70-J^a^	Nile tilapia	*S. iniae*	100	*S. iniae*	2.988	*S. agalactiae*	1.688
NS74-J^a^	Barramundi	*S. iniae*	100	*S. iniae*	2.878	*S. parauberis*	1.987
NS76-J^a^	Barramundi	*S. iniae*	99	*S. iniae*	2.999	*S. pyogenes*	1.555
NS185-J^a^	Barramundi	*S. iniae*	99	*S. iniae*	2.778	*S. parauberis*	1.654
NS11	Nile tilapia	*S. iniae*	98	*S. iniae*	2.376	*S. agalactiae*	1.997
NS18	Nile tilapia	*S. iniae*	100	*S. iniae*	2.419	*S. agalactiae*	1.707
NS26	Red tilapia	*S. iniae*	99	*S. iniae*	2.534	*S. pyogenes*	1.728
NS34	Barramundi	*S. iniae*	96	*S. iniae*	2.544	*S. agalactiae*	1.886
NS50	Barramundi	*S. iniae*	97	*S. iniae*	2.308	*S. pyogenes*	1.679
NS84	Barramundi	*S. iniae*	98	*S. iniae*	2.118	*S. pyogenes*	1.679
NS85	Barramundi	*S. iniae*	98	*S. iniae*	2.445	*S. agalactiae*	1.864
NS89	Red tilapia	*S. iniae*	99	*S. iniae*	2.342	*S. agalactiae*	1.975
NS90	Red tilapia	*S. iniae*	100	*S. iniae*	2.221	*S. agalactiae*	1.873
NS91	Nile tilapia	*S. iniae*	100	*S. iniae*	2.464	*S. parauberis*	1.593
*Aeromonas spp*							
A28-J^a^	Nile tilapia	*A. hydrophila*	100	*A. hydrophila*	2.978	*A. veronii*	1.495
A29-J^a^	Hybrid catfish	*A. hydrophila*	100	*A. hydrophila*	2.991	*A. veronii*	1.767
A49-J^a^	Red tilapia	*A. hydrophila*	100	*A. hydrophila*	2.965	*A. hydrophila*	1.869
A50-J^a^	Nile tilapia	*A. hydrophila*	100	*A. hydrophila*	2.889	*A. ichthiosmia*	2.094
A84-J^a^	Snakehead fish	*A. hydrophila*	100	*A. hydrophila*	2.945	*A. hydrophila*	1.755
A90	Nile tilapia	*A. hydrophila*	99	*A. hydrophila*	2.312	*A. veronii*	2.018
A108	Hybrid catfish	*A. hydrophila*	100	*A. hydrophila*	2.464	*A. hydrophila*	1.956
A109	Red tilapia	*A. hydrophila*	100	*A. hydrophila*	2.115	*A. hydrophila*	1.848
A110	Snakehead fish	*A. hydrophila*	98	*A. hydrophila*	2.394	*A. hydrophila*	2.181
A112	Snakehead fish	*A. hydrophila*	98	*A. hydrophila*	2.601	*A. hydrophila*	2.011
A114	Nile tilapia	*A. hydrophila*	97	*A. hydrophila*	2.451	*A. veronii*	1.995
A115	Nile tilapia	*A. hydrophila*	97	*A. hydrophila*	2.551	*A. ichthiosmia*	2.045
A120	Hybrid catfish	*A. hydrophila*	99	*A. hydrophila*	2.009	*A. veronii*	1.454
A126	Snakehead fish	*A. hydrophila*	100	*A. hydrophila*	2.567	*A. veronii*	1.777
A127	Snakehead fish	*A. hydrophila*	99	*A. hydrophila*	2.301	*A. shigelloides*	1.985
SB1-J^a^	Nile tilapia	*A. veronii*	100	*A. veronii*	2.969	*A. hydrophila*	1.559
SB2-J^a^	Nile tilapia	*A. veronii*	99	*A. veronii*	3.000	*A. shigelloides*	1.997
SB3-J^a^	Barramundi	*A. veronii*	99	*A. veronii*	2.897	*A. veronii*	1.787
SB4-J^a^	Barramundi	*A. veronii*	100	*A. veronii*	2.888	*A. veronii*	1.945
SB7-J^a^	Red tilapia	*A. veronii*	98	*A. veronii*	2.899	*A. shigelloides*	1.658
SB5	Nile tilapia	*A. veronii*	98	*A. veronii*	2.327	*A. ichthiosmia*	2.027
SB6	Nile tilapia	*A. veronii*	100	*A. veronii*	2.454	*A. hydrophila*	1.787
SB8	Barramundi	*A. veronii*	98	*A. veronii*	2.382	*A. hydrophila*	1.844
SB9	Barramundi	*A. veronii*	98	*A. veronii*	2.511	*A. veronii*	1.906
SB10	Barramundi	*A. veronii*	97	*A. veronii*	2.377	*A. veronii*	1.733
SB12	Barramundi	*A. veronii*	100	*A. veronii*	2.401	*A. veronii*	1.667
SB13	Barramundi	*A. veronii*	99	*A. veronii*	2.359	*A. hydrophila*	1.872
SB14	Barramundi	*A. veronii*	97	*A. veronii*	2.228	*A. ichthiosmia*	1.560
SB15	Barramundi	*A. veronii*	100	*A. veronii*	2.553	*A. veronii*	1.924
SB17	Barramundi	*A. veronii*	100	*A. veronii*	2.198	*A. veronii*	1.855
*Edwardsiella tarda*							
Ed10-J^a^	Hybrid catfish	*E. tarda*	100	*E. tarda*	2.899	*E. tarda*	2.000
Ed12-J^a^	Hybrid catfish	*E. tarda*	100	*E. tarda*	2.932	*E. tarda*	1.887
Ed14-J^a^	Nile tilapia	*E. tarda*	99	*E. tarda*	2.984	*E. tarda*	1.666
Ed16-J^a^	Nile tilapia	*E. tarda*	98	*E. tarda*	3.000	*E. hoshinae*	1.669
Ed18-J^a^	Nile tilapia	*E. tarda*	100	*E. tarda*	2.999	*E. hoshinae*	1.842
Ed8	Hybrid catfish	*E. tarda*	99	*E. tarda*	2.382	*E. hoshinae*	1.584
Ed9	Hybrid catfish	*E. tarda*	100	*E. tarda*	2.367	*E. hoshinae*	1.624
Ed11	Hybrid catfish	*E. tarda*	98	*E. tarda*	2.203	*E. hoshinae*	1.782
Ed17	Nile tilapia	*E. tarda*	99	*E. tarda*	2.471	*E. tarda*	1.977
Ed20	Nile tilapia	*E. tarda*	100	*E. tarda*	2.457	*E. tarda*	1.945
Ed23	Hybrid catfish	*E. tarda*	98	*E. tarda*	2.457	*E. tarda*	2.254
Ed25	Hybrid catfish	*E. tarda*	100	*E. tarda*	2.342	*E. tarda*	1.963
Ed27	Nile tilapia	*E. tarda*	100	*E. tarda*	2.264	*E. tarda*	2.014
Ed30	Nile tilapia	*E. tarda*	100	*E. tarda*	2.445	*E. tarda*	2.002
Ed31	Nile tilapia	*E. tarda*	100	*E. tarda*	2.116	*E. tarda*	1.956

^a^Isolate used for MSP database construction

log score values: < 1.700 = no reliable identification, ≥ 1.700–1.999 = probable genus-level identification, 2.000–2.229 = a secure genus-level identification and a probable species-level identification, and 2.300–3.000 = a secure species-level identification and highly probable species-level identification

**Table 2:** Comparison of the test results for bacterial species identification using a custom MSP database and Bruker database

Pathogen	MSP database	No. of isolate matched with 16 s rDNA sequencing (%)	Mean log score ± SD (Min - Max)
*S. agalactiae*	Custom	15/15 (100%)	2.599 ± 0.26 (2.244–2.999)
	Bruker	9/15 (60%)	1.758 ± 0.15 (2.545–2.019)
*S. iniae*	Custom	15/15 (100%)	2.554 ± 0.25 (2.118–2.999)
	Bruker	0/15 (0%)	1.753 ± 0.17 (1.432–1.997)
*A. hydrophila*	Custom	15/15 (100%)	2.568 ± 0.22 (2.009–2.991)
	Bruker	6/15 (40%)	1.883 ± 0.20 (1.454–2.181)
*A. veronii*	Custom	15/15 (100%)	2.562 ± 0.25 (2.198–3.000)
	Bruker	7/15 (46.67%)	1.808 ± 0.14 (1.559–2.027)
*E. tarda*	Custom	15/15 (100%)	2.554 ± 0.24 (2.116–3.000)
	Bruker	10/15 (66.67%)	1.877 ± 0.18 (1.584–2.254)
Total	Custom	75/75 (100%)	2.568 ± 0.25 (2.009–3.000)
	Bruker	32/75 (42.67%)	1.816 ± 0.17 (1.432–2.254)

## Discussion

An accurate and repeatable method for identification of the important bacterial pathogens of aquaculture species was established in this study. This method can be performed in a relatively short time compared to conventional microbiological methods. However, the present study found that reliable identification of bacterial species was only obtained when a custom MSP database was constructed since the reference database does not always accommodate the tested pathogens. Our study shows that identification was significantly improved when a custom MSP database was applied. All 75 isolates were secure at a genus-level identification and up to 88% (66 out of 75 isolates) were identified a highly probable species level when the identification was made on a custom MSP database. The public database may predominantly contain bacterial species that are only significant to humans but not species of veterinary importance, particularly from aquatic species [17]. The failure in species identification from the Bruker database may also result from inconsistent peptide profiles due to the use of different sample preparation protocols. The extraction method usually involves the use of organic acid to extract small-sized protein molecules, such as ribosomal proteins, cold shock proteins, and nucleic-acid binding proteins [18]. The different percentage of acid used in other studies [50% ACN and 2.5% TFA] [16, 19, 20] may alter the pattern of those extracted proteins. Nevertheless, the ability to tailor a database expands the application of MALDI Biotyper as an identification tool for bacterial species specific to a host or location, and at below species-levels, such as sub-species, strain, or serotype [21, 22].

The 3D-PCA scatterplot and MSP dendrogram generated from the MSPs can also be used for grouping or discriminating the type of organisms. The analyzed peptides are mainly ribosomal peptide molecules which uniquely present in the organisms [23]. In the present study, we provide an example of a MSP dendrogram created by the Biotyper software, by grouping the bacteria based on their phenotypic traits instead of their genetic traits (Fig. 2b). The software allows us to insert additional MSPs of other bacterial strains available in the reference database. Interestingly, the ATCC strains from the reference database are located in a different clade from our field strains. This may explain the failure of species identification described previously. Genotyping is usually based on phylogenetic analysis of a highly conserved region of the ribosomal RNA of the bacteria and this conservative feature may limit classification of the bacteria. Several studies have used MALDI-TOF MS as a discriminatory tool for typing bacterial pathogens [17, 24, 25] and have found that genotypic and phenotypic traits do not always concur [26]. Thus, MALDI-TOF MS can be used as an additional method for bacterial taxonomic classification when a complete MSP database is used, which may benefit further research, such as epidemiology, identification of protein biomarkers, and virulence studies. For example, MALDI-TOF MS has been used to distinguish antimicrobial resistant Enterobacteriaceae [27], identify *Burkholderia pseudomallei* mutants [18], and Carbapenem-resistant *Klebsiella pneumoniae* [28].

## Conclusions

To our knowledge, the present study is the first to describe a MSP database for both Gram-positive (*Streptococcus*) and Gram-negative (*Aeromonas* and *Edwardsiella*) bacterial pathogens of cultured fish. This analysis could establish species-level identifications, even when the sources of those bacteria are from different geographical locations or host species. However, this specificity can be obtained only when a custom MALDI Biotyper database is constructed with a standard sample preparation protocol. For the most reliable results, we suggest that the database of each user should contain a custom MSP of the active local pathogenic strains.

## Methods

### Bacterial samples

All bacterial isolates were obtained from clinical cases that were submitted for disease diagnosis at the Faculty of Veterinary Science, Chulalongkorn University, Thailand. Bacteriology was conducted by the methods described previously [4]. Diseased fish were dissected dorsoventrally with a sterile blade to expose the kidney. Bacterial isolation was then performed using a kidney swab onto Columbia blood agar supplemented with 5% sheep blood (Oxiod, Basingstoke, UK) and incubated at 28 °C for 24 h. A single colony of the pure (homogeneous colony appearance) bacterial culture on an agar plate was selected for species confirmation by conventional microbiology methods, including Gram staining, catalase and oxidase production tests, and API identification (BioMérieus®, France). Bacterial species were confirmed using PCR amplification and sequencing of the 16S rDNA [29]. All bacterial isolates were stored in a nutrient broth (NB; Oxiod) containing 10% fetal calf serum and 20% glycerol at -80 °C for further analysis.

### Sample preparation for MALDI-TOF MS

Each bacterial isolate was revived from the stock onto Columbia blood agar and incubated at 28 °C for 18 h. Extraction of bacterial proteins was performed as previously described [30]. A loopful of bacterial colonies was suspended in 70% ethanol and the suspension was centrifuged at 11,000 g for 2 min. The supernatant was removed, and the bacterial pellet was resuspended and mixed thoroughly with 100% acetonitrile (ACN) containing 5% (w/v) trifluoroacetic acid (TFA). The suspension was centrifuged and the supernatant was collected for peptide measurement using Lowry’s assay at 690 nm absorbance [31]. The concentration of peptide was adjusted to 0.1 μg μL^− 1^ for the MALDI-TOF MS analysis.

### MALDI-TOF MS for database generation

Five bacterial isolates of *S. agalactiae, S. iniae, A. hydrophila, A. veronii* and *E. tarda* were used as a representative for the MSP database preparation (Table 3). The peptide extraction was performed once for each bacterial isolate as described above. The protocol for database construction was referred to the previous study [32]. Peptide patterns of all isolates were identified by MALDI-TOF MS to generate a database specific to each bacterial species. The MALDI matrix solution [10 mg mL^− 1^ sinapinic acid in 100% ACN containing 5% (w/v) TFA] was added to each sample (0.1 μg μL^− 1^ peptide) at a 1:1 (v/v) ratio. The mixed samples were spotted and air dried onto a MTP 384 ground steel target plate (Bruker Daltonics, Billerica, MA, USA) as 29 individual replicates. Mass spectra were obtained using an Ultraflex III MALDI-TOF/TOF (Bruker Daltonik, GmbH, Germany) in a linear positive mode with a mass range between 2 and 20 kDa, a laser frequency of 50 Hz and 500 laser shots. A ProteoMass Peptide & Protein MALDI-MS Calibration Kit (Sigma Aldrich, MO, USA) was applied for external calibrations which consisted of human angiotensin II (m/z 1046), P14R (m/z 1533), human adrenocorticotropic hormone fragment 18–39 (m/z 2465), bovine insulin oxidized B chain (m/z 3465), bovine insulin (m/z 5731), and cytochrome C (m/z 12,362).

**Table 3:** Bacterial pathogens used for the development the custom MSP database. The isolates were obtained from cultured Nile tilapia *Oreochromis niloticus*, red tilapia *Oreochromis spp.*, barramundi *Lates calcarifer*, hybrid catfish *Clarias macrocephalus × C. gariepinus*, and Snakehead fish *Channa striata*

Bacterial species	Isolate number	Year	Source	Region
*S. agalactiae*	S147-J	2013	Nile tilapia	Western Thailand
	S183-J	2015	Red tilapia	Central Thailand
	S187-J	2017	Nile tilapia	Eastern Thailand
	S190-J	2018	Red tilapia	Southern Thailand
	SV1/1-J	2018	Nile tilapia	Northern Vietnam
*S. iniae*	NS12-J	2007	Red tilapia	Northeastern Thailand
	NS70-J	2012	Nile tilapia	Northeastern Thailand
	NS74-J	2014	Barramundi	Eastern Thailand
	NS76-J	2014	Barramundi	Eastern Thailand
	NS185-J	2018	Barramundi	Eastern Thailand
*A. hydrophila*	A28-J	2011	Nile tilapia	Eastern Thailand
	A29-J	2011	Hybrid catfish	Eastern Thailand
	A49-J	2013	Red tilapia	Central Thailand
	A50-J	2015	Nile tilapia	Eastern Thailand
	A84-J	2017	Snakehead fish	Central Thailand
*A. veronii*	SB1-J	2017	Nile tilapia	Eastern Thailand
	SB2-J	2017	Nile tilapia	Eastern Thailand
	SB3-J	2018	Barramundi	Eastern Thailand
	SB4-J	2019	Barramundi	Eastern Thailand
	SB7-J	2019	Red tilapia	Eastern Thailand
*E. tarda*	Ed10-J	2012	Hybrid catfish	Central Thailand
	Ed12-J	2013	Hybrid catfish	Central Thailand
	Ed14-J	2015	Nile tilapia	Eastern Thailand
	Ed16-J	2016	Nile tilapia	Eastern Thailand
	Ed18-J	2017	Nile tilapia	Central Thailand

Fingerprint spectra were calibrated and analyzed by the flexAnalysis software version 3.4 to assess high levels of reproducibility. The uniformity and homogeneity of the sample group as PMF and 3D-PCA were determined by t-test/ANOVA incorporated in the ClinProTools software version 3.0 [28]. A construction of a 3D-PCA scatterplot was performed using ClinProTools software.

The custom MSP database construction was performed according to Bruker’s recommendation. Twenty apparent spectra were chosen from MALDI-TOF analysis of each bacterial isolate, then a total of 100 spectra from 5 isolates of one bacterial pathogen were uploaded into MALDI Biotyper software (version 4.0) and assembled to generate a MSP database for the species using the standard method of BioTyper MSP creation. The MSP dendrogram was then created to determine the relatedness of each bacterial species based on their peptide fingerprint.

### Method validation

Reliability, repeatability and specificity of the method were evaluated by testing 15 bacterial isolates per bacterial species (Table 1). The bacteria were retrieved from -80 °C stock and processed through bacterial protein extraction, MAILDI-TOF MS, and species identification via BioTyper software using a similar protocol described above, each extracted sample was spotted as four replicates on the MALDI plate. The MSPs of these isolates were then blasted against the Bruker database and a custom MSP database. The reliability of the method was determined based on log score values computed by Biotyper software [33]; < 1.700 = no reliable identification (indicating inaccurate identification), ≥ 1.700–1.999 = probable genus-level identification, 2.000–2.229 = a secure genus-level identification and a probable species-level identification, and 2.300–3.000 = a secure genus-level identification and highly probable species-level identification. The ≤10% variation of log scores justified repeatability. Specificity was evaluated against the reference method, 16S rDNA sequencing. Numbers of the bacterial isolate that provided similar identification as detected by the reference method indicated a degree of specificity of the MALDI-TOF application.

## Data Availability

The datasets used and analyzed during the study are available from the corresponding author upon request.

## Abbreviations

3D-PCA: Three-Dimensional Principal Component Analysis
MALDI-TOF MS: Matrix-Assisted Laser Desorption/ionization Time-Of-Flight Mass Spectrometry
MSP: Main Spectra Profile
PMF: Peptide Mass Fingerprint
